# Can Simulated Green Exercise Improve Recovery From Acute Mental Stress?

**DOI:** 10.3389/fpsyg.2018.02167

**Published:** 2018-11-13

**Authors:** John James Wooller, Mike Rogerson, Jo Barton, Dominic Micklewright, Valerie Gladwell

**Affiliations:** School of Sport, Rehabilitation and Exercise Sciences, University of Essex, Colchester, United Kingdom

**Keywords:** green exercise, stress, mood, recovery, sensation, perception, nature, psychological

## Abstract

This exploratory study enhances previous research into green exercise and addresses a gap in the research by exploring the contribution of individual and combined senses in the recovery of mood and stress after a psychological stressor, whilst rigorously controlling exercise intensity. The hypotheses were: (i) recovery of mood and stress from a state of psychological stress would be greater following simulated green exercise compared to rest, (ii) green exercise would facilitate better recovery than exercise alone, (iii) these effects would remain 10 min following intervention, and (iv) visual stimuli alone would enhance recovery from a state of psychological stress compared to sound. Fifty participants were randomly assigned to one of five groups: REST, exercise, exercise with nature sounds, exercise with nature visual and exercise with nature sound and visual. An initial visit to obtain predicted peak power output values and to familiarize participants with the equipment being used was followed by a second visit, where participants experienced one test condition. Baseline measures of heart rate, blood pressure, total mood disturbance (TMD), and perceived stress were taken, before participants completed a stressor based on the Trier Social Stress test. Measures of heart rate and blood pressure were recorded in the last 30 s of the stressor to assess efficacy of the stressor. Immediately post stressor, measures of mood and perceived stress were taken followed by the intervention assigned (one of five described above). Measures of mood and perceived stress were taken again immediately post intervention and 10 min post intervention. Results showed that green exercise improved mood and stress scores more than exercise alone or REST. For both TMD and perceived stress, improvements in all simulated nature conditions were significantly improved compared to REST or exercise alone immediately post intervention. There were no significant changes 10 min post intervention in either mood or perceived stress compared to immediately post intervention values in any of the groups. This study suggests that environmental exercise settings including nature sounds, visual or both combined should be considered as important in the use of exercise as a therapeutic activity or recovery from acute psychological stress.

## Introduction

Psychological stress is defined as “a state of mental or emotional strain or tension resulting from adverse or demanding circumstances” (English Oxford Living Dictionaries [EOLD], 2018). Although stress tolerance varies between individuals due to the appraisal of the stressor, prolonged exposure to stress is considered a risk factor of poor health, due to the sustained physiological changes in response to the psychological demands (Mental Health Foundation [MHF], 2018). The psychological stress response is mediated by a cascade of hormones from the central nervous system and peripheral organs (Chrousos, 2009). Chronic psychological stress increases risk of health problems including cardiovascular, neurological, and mental ill health (including depression) (Oken et al., 2015).

Mental ill-health is one of largest factors in global disease burden, with depression the leading cause of disability (Vos et al., 2015). Each year in the United Kingdom, around 12 million adults seek medical advice about their mental health, many relating to anxiety and depression, which are often associated with, or triggered by, high levels of stress (Mental Health Foundation [MHF], 2018). In 2016/17 work-related stress alone was responsible for 12.5 million lost work days in the United Kingdom, accounting for half of all absences due to ill health (Health Safety Executive [HSE], 2017). Longitudinal studies and systematic reviews have indicated that work-related stress is associated with anxiety, depression, heart disease and some musculoskeletal disorders (Health Safety Executive [HSE], 2017). A clearer understanding of the interventions that ameliorate stress and enhance recovery is needed (Danielsson et al., 2012), especially given the wider negative consequences it has on individual health, society and the economy (Health Safety Executive [HSE], 2017).

Nature and green environments contribute to an enhanced level of physical and mental health (Ward Thompson et al., 2012; Gladwell et al., 2013; Hartig et al., 2014; Pearson and Craig, 2014; Akpinar et al., 2016; van den Berg et al., 2016; Douglas et al., 2017; Ekkel and de Vries, 2017; Wood et al., 2017; Hazer et al., 2018). Over the last decade, epidemiological studies have shown positive associations between quantity of local green space and improved health outcomes (Mitchell and Popham, 2008; Maas et al., 2009; Beyer et al., 2014; Kardan et al., 2015; Ward Thompson et al., 2016). Being in green spaces may relieve stress since lower perceived stress has been associated with greater weekly exposure to green spaces (Hazer et al., 2018). Thus, links between engagement with green spaces and wide-ranging health benefits have become a focal point for research.

It has been suggested that modern day humans have an innate connection with nature and living things due to our hunter-gatherer past (Kellert and Wilson, 1995). Natural environments can be enjoyed without having to deliberately focus attention, concentrate or expend mental effort. This has led some to claim exposure to nature has restorative effects on mental fatigue and attention (Kaplan, 1995; Berman et al., 2008). Nature and natural environments have been found to counteract the negative effects of stress, specifically with respect to stress recovery (Brown et al., 2013), mental fatigue reduction (Berman et al., 2008, 2012; Taylor and Kuo, 2009), and cognitive restoration (Kaplan, 1995; Grahn and Stigsdotter, 2003; Berto, 2005; Bowler et al., 2010; Rogerson and Barton, 2015).

Direct contact with nature is not necessary for it to facilitate recovery from stress. Viewing nature through a window (Ulrich, 1984; Kaplan, 2001), by means of still or moving images projected onto a screen (Brown et al., 2013; Wooller et al., 2015), and through virtual reality (Annerstedt et al., 2013) have all improved recovery from acute stress. Viewing images of nature 10 min prior to being subjected to an acute mental stressor was sufficient to positively affect the recovery of the autonomic system (Brown et al., 2013). Recovery from a virtual reality version of the Trier Social Stress Test (TSST) was found to be best when exposed to a simulated natural environment comprising both sounds and images, rather than just images of nature or a control condition absent of all nature images and sounds (Annerstedt et al., 2013). Using similar sensory isolation methods combined with moderate intensity cycling, positive effects on mood were found when the simulated green environment included both video graphic and auditory components (Wooller et al., 2015). Unexpectedly, the largest mood improvement occurred when the sounds of nature were excluded from the simulation compared to the removal of the sight or smell of nature (Wooller et al., 2015).

Exercise performed in conjunction with exposure to nature is known as green exercise (Pretty et al., 2005) and has been associated with a variety of psychological and physiological benefits (White et al., 2013; Weng and Chiang, 2014). Green exercise improves mood, attention and physiological markers such as heart rate, blood pressure and cortisol compared to exercise in built man-made environments (Focht, 2009; Li et al., 2011; Thompson Coon et al., 2011; Rogerson and Barton, 2015). While these and other effects of green exercise are well documented, less is known about which senses might have the greatest contribution to the reported outcomes. Previous green exercise research showing beneficial effects on attention and psychological recovery (Focht, 2009; Li et al., 2011; Thompson Coon et al., 2011; Rogerson and Barton, 2015) can be furthered by investigating in more detail the contribution of individual senses and multi-sensory integration in situations where a state of stress has been intentionally induced. Using simulated green exercise in a laboratory environment minimizes less controllable variables such as the weather, terrain and contact with other people, whilst enabling control of the exercise intensity, mode and stimulated senses.

The purpose of this exploratory study was to investigate the effects of simulated green exercise used as a recovery intervention following exposure to acute mental stress on immediate mood and stress levels and whether any recovery effects persisted following a further 10 min of rest. Additionally, to explore the influence of visual and auditory senses, these senses were manipulated to allow sight or sound to be the main contributing sense during the green exercise simulation. The olfactory sense was excluded for this study as previous work showed that smell had a limited impact on the green exercise outcomes (Wooller et al., 2015). The hypotheses were that: (i) recovery of mood and stress from a state of psychological stress would be greater following simulated green exercise compared to resting recovery, (ii) simulated green exercise would facilitate better recovery compared to exercise alone, (iii) these effects would remain 10 min following simulated green exercise, and (iv) visual stimuli alone would enhance recovery of mood and stress from a state of psychological stress compared to sound.

## Materials and Methods

### Participants

Fifty healthy participants were recruited for this study (Age 27.2 ± 10.2 years; Stature 173.8 ± 9.1 cm; Body Mass 78.3 ± 16.4 kg; Body Mass Index 25.8 ± 4.7 kg.m^2^) constituted of 34 males (Age 25.7 ± 9.5 years; Stature 178.4 ± 6.2 cm; Body Mass 83.3 ± 15.8 kg; Body Mass Index 26.2 ± 4.9 kg.m^2^) and 16 females (Age 30.4 ± 11.3 years; Stature 164.2 ± 6.1 cm; Body Mass 67.5 ± 11.9 kg; Body Mass Index 25.0 ± 4.3 kg.m^2^). Only healthy individuals free from chronic conditions, injury and illness were permitted to take part, this was verified by use of a physical activity readiness questionnaire (PAR-Q). Written informed consent was provided by all participants and the study and its associated procedures were approved by the University of Essex ethics committee.

### Design

A between-subjects experimental design was used in which participants attended the laboratory on two occasions. The first visit was to establish participants estimated peak power output (EPPO) using a CatEye ergociser (EC-1600, CatEye Co., Ltd., Osaka, Japan). On the second visit participants were randomly allocated to one of five stress recovery groups: (i) Rest, (ii) Cycling without nature simulation, (iii) Cycling with simulated nature sounds, (iv) Cycling with simulated nature video, or (v) Cycling with simulated nature sounds and video combined. Quota sampling methods were used to ensure an even number of participants (*n* = 10) per condition. Participants were not aware of their grouping prior to the recovery intervention. Further, the tester inducing the stress was not aware of the group the participant was in.

**FIGURE 1:**
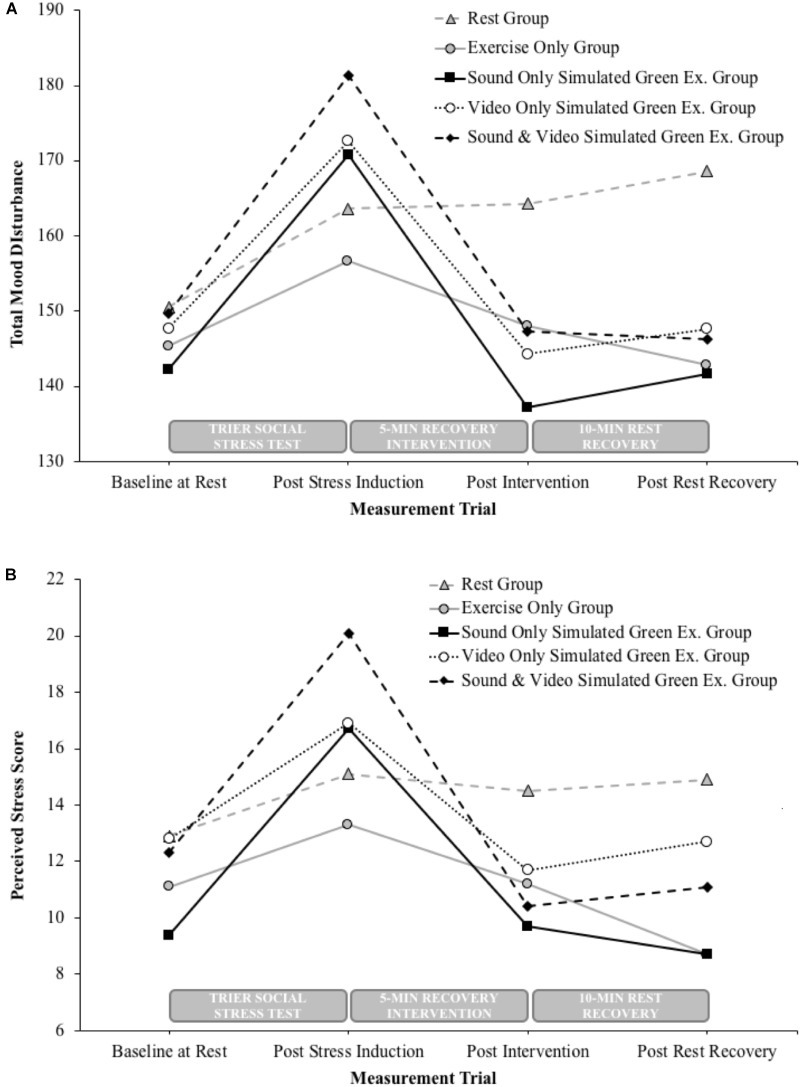
Between group changes in total mood disturbance **(A)** and perceived stress **(B)** following the stress induction task, 5-min recovery intervention and 10-min resting recovery.

During the second visit, participants carried out a stress induction task (described in Stress Induction) followed by 5 min of moderate intensity cycling under the simulated green exercise conditions associated with the condition they had been assigned to (see Stress Recovery Interventions). A variety of dependent variables were recorded including mood, perceived stress, heart rate and blood pressure. All measurements were taken before and after the stress induction task. Mood and perceived stress were also taken immediately after the green exercise cycling task, and 10 min after resting recovery. The measurement trials in relation to the stress induction task, recovery intervention and further 10 min rest period are indicated above the *x*-axis on Figure 1.

### Cycling Ergometry

During the first visit, EPPO was calculated using the YMCA bicycle submaximal fitness test (Golding et al., 1989) programmed into a CatEye ergociser as used by Rogerson et al. (2016). During the experimental conditions, a 100p/100k Ergoselect cycle ergometer (Ergoline, Bitz, Germany) was used. The Ergoselect allowed stringent control of exercise intensity, by continually adjusting pedaling cadence to maintain constant intensity wattage. Exercise intensity was set at 40% EPPO, in accordance with previous methods used to replicate moderate exercise (Wooller et al., 2015).

### Stress Induction

Each participant individually carried out a TSST in accordance with the methods of Kirschbaum et al. (1993). Participants were first taken into a plain room where two testers, seated behind a table, explained the test. Participants were instructed to stand on a marker positioned on the floor in front of the testers which they were told was necessary for video capture purposes. Participants were bought into the room at a time when they could see one of the testers adjusting the camera equipment, which was visible from the marker position. At the end of all testing, participants were debriefed that in fact no recordings were made, and that the presence of the camera was intended to add to their stress. The testers explained to participants that they would be required to complete a mathematics and English task but provided no further details. Participants were then invited to wait outside of the room and permitted 5 min to mentally prepare themselves for the upcoming tasks.

After 5 min, participants were bought back into the room. The testers were instructed to show no signs of emotion or assist the participants in anyway. One tester administered a mathematics task, which required the participant to count backward by 13 from 1677. In the event of a mistake, a loud beep was sounded, and the participant was instructed to start again from 1677. The second tester administered an English task, which required participants to spell words, ranging from seven to ten letters long, backward. Again, in the event of a mistake a loud beep was sounded, and the participant was asked to spell that word again. Each task lasted for 5 min and participants were randomly assigned to order counterbalanced tasks.

### Stress Recovery Interventions

Each participant performed one of five stress recovery interventions according to the condition they had been randomly assigned. Standardization of the recovery environment, to minimize confounding or extraneous effects on the dependent variables, was achieved by having participants complete all conditions in identical laboratory settings, seated on a cycling ergometer positioned in front of a projector screen. All recovery interventions lasted for 5 min which has previously been found sufficient for green exercise effects to occur (Barton and Pretty, 2010; Wooller et al., 2015).

Participants in the rest condition sat quietly on the cycle ergometer in front of a gray screen. During exercise without simulated nature, participants cycled at 40% EPPO in front of a gray screen. In the three remaining simulated nature cycling conditions, participants cycled at 40% either a gray screen and the soundtrack of birdsong (simulated nature sounds only), video images of nature but no sounds played (simulated nature scenes only) or while both the simulated sounds and video images of nature were presented.

Nature sounds and images were taken from a commercially available exercise DVD (Fitness Journeys – through the forest, Isis Asia Ltd., Manila, Philippines) and projected onto a large screen positioned approximately 150 cm in front of the participant. Video image size was 180.3 cm × 92.5 cm and 126 cm from the ground. To ensure an environment where no other people or moving vehicles were present, the last 5 min of the DVD chapter “Redwoods and Oaks” was used. Playback speed simulated moving at approximately 20 km.hr^-1^ which, together with the proximity of the screen to the participant, gave a realistic simulated cycling experience of forward movement. This DVD and screen set up had been used in our laboratories in a previous study conducted (Wooller et al., 2015).

Dependent variables were captured immediately after each stress recovery intervention and then participants were asked to rest in silence in front of a gray projector screen while remaining seated on the cycle ergometer for a further 10 min. Dependent variables were recorded again at the end of the 10 min rest period.

### Psychological and Physiological Measurements

#### Mood State

The shortened “right now” version of the Profile of Mood States (POMS) questionnaire (McNair et al., 1971, 1992) was used to measure mood. This version uses a 30-point item, scored using a five-point Likert scale ranging from “0 = Not at all” to “4 = Extremely.” Subscale scores for Tension, Depression, Anger, Vigor, Fatigue and Confusion were calculated. Total mood disturbance (TMD) was then calculated by subtracting the vigor score for from the sum of the other five subscales. This gave an overall value for TMD between 112 and 282, giving an indication of overall mood with higher TMD suggesting lower mood. POMS was measured four times: (i) baseline on arrival; (ii) immediately after the stress induction task; (iii) immediately after the recovery intervention, and (iv) after 10 min of rest.

#### Stress Measures

Stress was measured using the Perceived Stress Scale (PSS) (Cohen et al., 1983; Cohen and Williamson, 1988). PSS comprises ten statement items to measure an individual’s self-appraisal of how potentially stressful their life is (Cohen and Williamson, 1988). A modified version of the ten item PSS was used, in accordance with (Rogerson et al., 2015), to measure ‘right now’ state measurements of perceived stress. Item statements such as ‘In the last month, how often have you been upset because of something that happened unexpectedly?’ was edited to say ‘I feel upset,’ with an accompanying instruction asking participants to ‘indicate how you feel right now, at this moment.’ On the original PSS responses were made using a Likert scale scored from 0 – ‘Never’ to 4 – ‘Very Often.’ The modified PSS used descriptors instead from 0 – ‘Strongly Disagree’ to 4 – ‘Strongly Agree.’ The range of aggregated scores was 0–40 with higher scores indicate a greater level of stress. PSS was administered at the same time points as POMS described above.

#### Heart Rate and Blood Pressure Measures

Heart rate (HR) and blood pressure (BP) were recorded at baseline and throughout the stressor using a Mobil-O-Graph 24 h PWA Monitor (I.E.M. GmbH, Stolberg, Germany) to establish physiological. The recorder was set to measure HR and BP every 2 min, the minimum time interval available (only data for the last 30 s of the stressor was used).

### Statistical Analysis

A manipulation check was carried out using a series of mixed two-way (5 × 2) ANOVAs to test whether the stress induction task had actually provoked negative changes in heart rate, blood pressure, mood and perceived stress as intended. The between-subjects factor was the recovery condition participants were assigned to, and the within-subjects factor was the measurement trial (pre- versus post-Trier Social Stressor measurement).

Total mood disturbance and PSS changes following the 5 min stress recovery intervention and 10 min rest period were analyzed using mixed two-way (5 × 3) ANOVAs. The between-subjects factor was the recovery condition participants were assigned to, and the within-subjects factor was the measurement trial (post stress induction task, post stress recovery intervention and post 10 min recovery). Two-way (5 × 3) ANCOVAs, using baseline scores as a covariate, were used to examine mood and PSS changes once individual variation in acute stress responses had been controlled for.

An alpha level of 0.05 was used to indicate statistical significance in all ANOVA and ANCOVA tests and where sphericity assumptions were violated, Greenhouse-Geisser outcomes are reported as indicated by adjusted degrees of freedom. Significant interactions were followed up using *post hoc* paired samples *t*-tests separately for each group to examine changes in mood and perceived stress before and after the recovery intervention, and after the 10 min rest recovery period. A Bonferroni corrected alpha level of 0.013 was used to indicate significance. Effect sizes are reported as eta-squared (*η^2^*) and partial eta-squared (η*_p_*^2^). All data analysis was conducted using SPSS v 24 (IBM Inc., New York NY, United States).

## Results

### Missing Data Imputation

Of the 50 participants, three (6%) had missing data. TMD data for all four trials were complete, however, among the PSS data there was one response missing from the post stress induction trial and two responses missing from the post recovery intervention trial equating to total missing PSS data of 1.5% (3/200).

Missing items were filled using iterative Markov Chain Monte Carlo multiple imputation methods incorporating linear regression to scale variables using a maximum of 10 iterations. The imputation model was constrained to produce integers only within the possible PSS minimum and maximum score range of 0 to 40, respectively, ensuring the imputed values corresponded with the PSS response scoring system. All missing data was resolved, and the resultant imputed dataset was used for all further analysis.

### Manipulation Check of the Trier Social Stress Test

The TSST provoked changes in heart rate (*F*_1,42_ = 29.7, *P* < 0.0001, η*_p_*^2^ = 0.41); systolic blood pressure (*F*_1,42_ = 44.4, *P* < 0.0001, η*_p_*^2^ = 0.51); diastolic blood pressure (*F*_1,42_ = 97.3, *P* < 0.0001, η*_p_*^2^ = 0.70); TMD score (*F*_1,45_ = 33.0, *P* < 0.0001, η*_p_*^2^ = 0.42); and PSS score (*F*_1,43_ = 47.2, *P* < 0.0001, η*_p_*^2^ = 0.49). As indicated in Table 1, all dependent variables significantly decreased, apart from TMD which increased (i.e., a decrease in mood) (*P* < 0.001). This indicates that a raised state of acute stress had been induced as intended.

**Table 1:** Changes in total mood disturbance and perceived stress following the strees induction task, 5 min recovery intervention and 10 min resting recovery.

	Baseline	Post stressor		Post intervention		Post 10-min rest recovery		
	Absolute values	Absolute values	Δ From baseline	Absolute values	Δ From post stressor	Absolute values	Δ From post stressor
**Total mood disturbance**		
Rest	150.5 ± 19.8	163.6 ± 28.7^∗^	13.1 ± 30.4	164.3 ± 29.6	0.7 ± 12.4	168.6 ± 32.4	5.0 ± 14.5
Exercise only	145.3 ± 19.6	156.6 ± 26.4^∗^	11.3 ± 14.0	148.0 ± 25.2	–8.6 ± 16.2	142.8 ± 15.7^†^	–13.8 ± 13.7
sound only	142.3 ± 15.8	170.8 ± 36.1^∗^	28.5 ± 24.1	137.2 ± 13.2^†^	–33.6 ± 24.3	141.7 ± 21.4^†^	–29.1 ± 25.0
Video only	147.7 ± 13.1	172.6 ± 23.9^∗^	23.0 ± 16.4	144.3 ± 12.0^†^	–28.3 ± 16.7	147.6 ± 15.4^†^	–25.0 ± 18.5
Sound and video	149.6 ± 18.4	181.3 ± 36.9^∗^	34.6 ± 43.1	147.3 ± 16.3^†^	–34.0 ± 36.7	146.3 ± 20.5^†^	–35.0 ± 24.9
**Perceived stress**
Rest	12.9 ± 5.7	15.1 ± 6.7^∗^	2.3 ± 5.2	14.5 ± 6.7	–0.6 ± 4.6	14.9 ± 7.6	–0.2 ± 4.2
Exercise only	11.1 ± 8.4	13.3 ± 8.0^∗^	3.2 ± 3.4	11.2 ± 8.1	–2.6 ± 4.1	8.7 ± 7.1^†^	–4.6 ± 3.0
Sound only	9.4 ± 5.8	16.7 ± 10.1^∗^	6.6 ± 7.9	9.7 ± 5.6^†^	–7.1 ± 6.8	8.7 ± 6.3^†^	–8.0 ± 9.2
Video only	12.8 ± 4.3	16.9 ± 2.5^∗^	4.1 ± 4.1	11.7 ± 1.9^†^	–5.2 ± 2.9	12.7 ± 2.3^†^	–4.2 ± 3.8
Sound and video	12.3 ± 3.4	20.1 ± 4.1^∗^	3.2 ± 5.4	10.4 ± 4.7^†^	–8.4 ± 8.1	11.1 ± 4.3^†^	–7.7 ± 6.7


*^∗^Indicates a significant increase post stressor compared to baseline (*P* < 0.0001); ^†^indicates significant reduction compared to post stress induction outcomes. All outcomes reported as mean ± 1 SD.*

### Recovery of Total Mood Disturbance

A two-way (5 × 3) ANOVA revealed an interaction effect between the intervention group and post stress task trial changes in TMD. This was accompanied by a trial main effect but no group main effect. Controlling for baseline TMD using a two-way (5 × 3) ANCOVA, produced a similar strength group-by-trial interaction, however, the trial main effect, although still significant, was diminished. Statistical outcomes are reported in Table 2.

**Table 2:** Effect differences in post intervention TMD and PSS outcomes when baseline stress has (ANCOVA) and has not (ANOVA) been controlled for.

	Total mood disturbance				PSS			
	*F*	(df)	*P*	ηp2	*F*	(df)	*P*	ηp2
**Trial main effects**								
ANOVA	38.0	(1.6, 70.3)	<0.0001	0.46	36.0	(1.6, 71.6)	<0.0001	0.45
ANCOVA	7.0	(1.5, 68.3)	0.004	0.14	0.3	(1.6, 70.8)	0.76	0.01
**Group main effects**								
ANOVA	0.9	(4, 45)	0.50	0.07	0.9	(4, 45)	0.46	0.08
ANCOVA	0.4	(4, 44)	0.78	0.04	0.7	(4, 44)	0.60	0.06
**Trial-by-group interactions**								
ANOVA	4.1	(6.2, 70.3)	0.001	0.27	4.6	(6.4, 71.6)	0.0004	0.29
ANCOVA	4.0	(6.2, 68.3)	0.002	0.27	4.8	(6.4, 70.8)	0.0002	0.31


*Non-integer degrees of freedom (df) values indicate use of Greenhouse-Geisser outcomes. Partial eta-squared (η*_p_*^2^) effect sizes are reported.*

*Post hoc* analyses showed reductions in TMD after 5 min of cycling among the nature sound group (*t*_9_ = 4.4, *P* = 0.001, *η*^2^ = 0.68, 95% CI = 16.2–51.0), nature video group (*t*_9_ = 5.4, *P* < 0.0001, *η*^2^ = 0.76, 95% CI = 16.4–40.2), and the combined nature sounds and video group (*t*_9_ = 2.9, *P* = 0.009, *η*^2^ = 0.49, 95% CI = 7.8–60.2). Over a subsequent 10 min resting recovery period, there was no further significant TMD change among the sound group (*t*_9_ = -0.8, *P* = 0.222, *η*^2^ = 0.07, 95% CI = -17.2–8.2), video group (*t*_9_ = -1.2, *P* = 0.136, *η*^2^ = 0.13, 95% CI = -9.7–3.1) or combined sound and video group (*t*_9_ = 0.2, *P* = 0.43, *η*^2^ < 0.01, 95% CI = -11.3–13.4). There was no significant TMD change in the exercise only group or the rest group following the initial 5 min recovery intervention period, however, compared to the post stressor measurements the exercise only group did exhibit lower TMD over a subsequent 10 min resting recovery period (*t*_9_ = 3.2, *P* = 0.006, *η*^2^ < 0.53, 95% CI = 4.0–23.6). Mean changes in TMD are given in Table 1 and presented in Figure 1A.

### Recovery of Perceived Stress

A two-way (5 × 3) ANOVA revealed an interaction effect between the intervention group and post stress task trial changes in PSS. This was accompanied by a trial main effect but no group main effect. Controlling for baseline PSS using a two-way (5 × 3) ANCOVA, produced a similar strength group-by-trial interaction, however, the trial main effect, although still significant, was diminished. Statistical outcomes are reported in Table 2.

*Post hoc* analyses showed reductions in PSS after 5 min of cycling among the nature sound group (*t*_9_ = 3.2, *P* = 0.005, *η*^2^ = 0.54, 95% CI = 2.1–11.9), nature video group (*t*_9_ = 5.8, *P* < 0.0001, *η*^2^ = 0.79, 95% CI = 3.2–7.2) and the combined nature sounds and video group (*t*_9_ = 4.5, *P* = 0.001, *η*^2^ = 0.69, 95% CI = 4.8–14.6). Over a subsequent 10 min resting recovery period, there was no further significant PSS change among the sound group (*t*_9_ = 0.7, *P* = 0.248, *η*^2^ = 0.05, 95% CI = -2.2–4.2), video group (*t*_9_ = -1.3, *P* = 0.115, *η*^2^ = 0.16, 95% CI = -2.8–0.8) or combined sound and video group (*t*_9_ = -0.7, *P* = 0.26, *η*^2^ = 0.05, 95% CI = -3.1–1.7). There was no significant PSS change in the exercise only group or the rest group following the initial 5 min recovery intervention period, however, compared to the post stressor measurements the exercise only group did exhibit lower PSS (*t*_9_ = 4.8, *P* = 0.001, *η*^2^ < 0.72, 95% CI = 2.4–6.8). Mean changes in PSS are given in Table 1 and presented in Figure 1B.

## Discussion

A key finding of this exploratory study is that all variations of simulated green exercise were more effective than both rest and indoor cycling at recovering from an episode of induced acute stress. A further, important contribution this study makes, is to extend our understanding of the sensory basis of green exercise, a critical early step in trying to move toward more explanatory, mechanistic models. Since the senses are first in the cognitive information processing cascade, an important finding of the present study is that green exercise simulations involving visual feedback during cycling appear to have the strongest impact on mood and perceived stress recovery. It was also found that the positive states of recovery observed in all green exercise conditions, and to a lesser extent in the non-green exercise condition, were preserved during a subsequent 10 min rest period.

### Green Exercise and Stress Recovery

The method of inducing an acute stress response that we used was effective as indicated in the significant increases in heart rate, blood pressure, mood disturbance and perceived stress. Inducing acute stress in this way is an important development in green exercise research because it carries high ecological validity in the sense that, owing to the complex array of stressors prevalent in contemporary society (Health Safety Executive [HSE], 2017; Mental Health Foundation [MHF], 2018), it is not uncommon for individuals to frequently experience sudden episodes of intense stress. In this context, our findings that green exercise facilitated recovery of mood and perceived stress quicker compared to those resting or exercise alone, has several important implications.

The first is that, notwithstanding the known barriers to readily accessing natural environments (Dahmann et al., 2010; Sister et al., 2010; Jennings et al., 2012), green exercise is an option that individuals may choose to quickly and effectively cope with stress. Consistent with previous findings (Taylor and Kuo, 2009; Barton and Pretty, 2010; Brown et al., 2013), we also found that green exercise was effective after just 5 min which adds to its viability as a coping strategy, particularly among those for whom the availability of time is a contributory stressor. For instance, those working in stressful environments with limited time to break such as teachers, drivers, construction workers, health professionals and many others.

The second important implication is that, as previously suggested (Barton and Pretty, 2010; Thompson Coon et al., 2011), our results indicate that experiencing nature can further enhance the psychological effects of exercise. Specifically, we observed improvements in TMD and PSS immediately following simulated green exercise conditions that were of a magnitude not seen in the exercise only condition. It is not that exercise is not effective but rather, as illustrated in Figure 1, seems to have a more gradual recovery course compared to the apparent immediate effects of green exercise. After only 5 min of green exercise, mood and perceived stress had, fallen back to baseline levels with just one exception, perceived stress in the simulated nature sound condition (Table 1). Interestingly, there appears to be a continued downward trend in both TMD and PSS in the 10-min post intervention suggesting a longer time period following the intervention should be explored, to better understand the enduring benefits of a single exposure to green exercise.

Controlling for variations in baseline mood and perceived stress only slightly dampened the interaction between recovery intervention and therapeutic effects (Table 2), and the *post hoc* analysis revealed very high effect sizes for all green exercise conditions. Consequently, we are able to report with high confidence, that green exercise was the best of all interventions we tested in recovering from acute stress.

### Sensory Factors in Green Exercise and Stress Recovery

Green exercise undertaken outdoors has multi-sensory aspects (Franco et al., 2017). Simulating green exercise enabled exploration of the relative influence of visual and auditory stimuli on green exercise recovery from acute stress. Large effect sizes were found in all green exercise conditions indicating that nature simulations involving isolated auditory feedback, isolated visual feedback and combined audio-visual feedback are all effective in recovering from acute stress. Isolated visual feedback was found to have the greatest influence on mood and perceived stress, with very large effect sizes of >0.75 measured in both instances. This is not surprising given that vision is considered to be the dominant sense, as demonstrated in classic studies of the ventriloquist effect (Thurlow and Jack, 1973; Warren et al., 1981) and McGurk effect (McGurk and MacDonald, 1976). Studies exploring the benefits of nature have mainly focused on visual aspects (Franco et al., 2017), however, our previous study that occluded nature stimuli found removal of sound to have the greatest impact on mood in comparison to removal of visual cues (Wooller et al., 2015).

In the current study, it is less clear is why the green exercise effect for vision alone was stronger (according to effect size) than combined audio-visual simulation of nature. Counterintuitively, it appears that the compound effects of audio-visual simulation are not as strong a visual input alone. This is unexpected given that audio-visual simulation is arguably more realistic than those simulation involving isolated audio or visual sensory inputs. A potential explanation might be found in the known complexities of cross-modal interactions on perception (Shams and Kim, 2010). Auditory emotional cues have, as the net result of competing task-relevant emotional priming and divided audio-visual attention demands, been found to enhance the processing of target visual information (Zeelenberg and Bocanegra, 2010). In the context of the green exercise simulations used in our study, the resultant effects on mood and perceived stress may therefore be due to the extent to which demands on attention compete with the cues from other senses. Since limited attentional capacity is divided in the audio-visual simulation, this might account for why the effect size was weaker compared to the isolated visual and auditory sensory conditions.

Another interesting and relevant body of work concerns cross-modal perceptual plasticity where enhanced sensory compensation has not only been found in those with visual or hearing impairments (Cecchetti et al., 2016) but also in those temporarily impaired, for instance through the use of a blindfold (Lee and Whitt, 2015). Cross-modal perceptual plasticity may in fact help explain why the effects were so strong in the isolated sensory conditions of our experiment, where auditory nature cues might have triggered relevant associated mental imagery of nature and vice-versa as previously reported (De Volder et al., 2001). What is clear is that, as our findings highlight, the sensory and perceptual mechanisms of the green exercise effect are most likely a product of complex cross-modal interactions and sensory compensatory processes that warrant further detailed investigation.

### Future Directions

The current study contributes to the growing body of research that has shown the use of green exercise, as an intervention when either physical or psychological systems have been negatively affected, to be a beneficial factor in recovery (Tsunetsugu et al., 2007; Barton and Pretty, 2010; Thompson Coon et al., 2011; Gladwell et al., 2013). It also adds to the previous research into the mechanisms of green exercise effect by identifying the role of individual and combined senses (Alvarsson et al., 2010; Saadatmand et al., 2013; Aghaie et al., 2014; Rogerson and Barton, 2015; Wooller et al., 2015). We suggest that the use of nature sounds and sights in conjunction with exercise may well promote the recovery of TMD and PSS after a stressor. This could aid in the development of cost-effective stress reducing strategies both in the workplace and personal life. It is important, however, to establish in future studies how long the effects may be sustained, what constitutes the best “stimulus” for stress recovery and who might benefit. Future study designs should also consider the level of connectedness to nature participants have prior to starting the study (Mayer and Frantz, 2004; Capaldi et al., 2014). This would further current understanding of how different individuals may benefit from green exercise participation. Certainly, future green exercise studies should include exploration of the use of virtual reality as it can offer more immersive experiences than currently achieved within current laboratory studies, but still allows control of confounding factors. Multi-sensory and non-sensory elements should be included where possible. Further, green exercise should be conducted in “real” natural spaces, with different duration and types of exposure, e.g., including level of engagement with nature, in a range of different cohorts. Outcome measures should be recorded for over 24 h.

## Conclusion

Exercise combined with nature, in whole or in part, can facilitate recovery of mood and perceived stress after an acute psychological stressor. The results indicate that exercise with nature sounds, nature visual or exercise with both nature sounds and visual are better for recovery from an acute stressor than rest or exercise alone, as shown by measures taken immediately post intervention and 10-min post intervention. Future work is required to explore the importance and mechanisms of each of the senses during exercise in contributing to improvements in TMD and PSS following a stressor. Overall, these results indicate that, environmental exercise settings which include nature sounds, visual nature or nature sounds with visual nature should be considered when using of exercise as a recovery from acute psychological stress and could be restorative of positive emotions which may help to buffer stress.

## Ethics Statement

This study was carried out in accordance with the recommendations of University of Essex ethics committee. The protocol was approved by the Faculty of Science and Engineering ethical committee at the University of Essex. All subjects gave written informed consent in accordance with the Declaration of Helsinki.

## Author Contributions

JW contributed to the concept, design, data collection, and writing of this research manuscript. MR contributed to the data analysis and writing of this research manuscript. JB contributed to the concept and writing of this research manuscript. DM contributed to the concept, design, data analysis, and writing of this research manuscript. VG contributed to the concept and design and writing of this research manuscript.

## Conflict of Interest Statement

The authors declare that the research was conducted in the absence of any commercial or financial relationships that could be construed as a potential conflict of interest.
